# Analytical validation of digital measure of intelligibility in progressive supranuclear palsy

**DOI:** 10.3389/fnins.2026.1961535

**Published:** 2026-09-28

**Authors:** İlkay Yıldız Potter, Ram Kinker Mishra, Tejas Pawar, Jesse Wang, Antje S. Mefferd, Michael de Riesthal, Mili Kuruvilla-Dugdale, Anne-Marie Wills, Alexander Pantelyat, Ashkan Vaziri

**Affiliations:** 1 BioSensics LLC, Newton, MA, United States; 2 Department of Neurology, Massachusetts General Hospital, Harvard Medical School, Boston, MA, United States; 3 Vanderbilt University Medical Center, Nashville, TN, United States; 4 Department of Communication Sciences and Disorders, University of Iowa, Iowa City, IA, United States; 5 Department of Neurology, Johns Hopkins University School of Medicine, Baltimore, MD, United States

**Keywords:** digital health, dysarthria, intelligibility, progressive supranuclear palsy, speech analysis and processing

## Abstract

**Introduction:**

Progressive supranuclear palsy (PSP) causes progressive speech impairment. Digital speech analysis can objectively and remotely quantify measures like intelligibility, typically judged subjectively by experts during clinical visits. The objective of this study is to validate digital intelligibility measurement in PSP via BioDigit Speech (BioSensics LLC, United States).

**Methods:**

Forty-nine PSP patients performed standardized passage-reading scored by BioDigit Speech and three experts (18 + years of experience); expert mean score (EMS) served as the reference standard.

**Results:**

Digital intelligibility showed high correlation and agreement with EMS (*r* = 0.90, ICC = 0.84 in native speakers), while systematically scoring below EMS. Moreover, the digital measure discriminated PSP clinical severity as well as or better than EMS, particularly for PSP-QoL-11 with a steeper standardized response. Correlations of digital measure with the clinical scales were moderate (up to −0.51), significant (*p*-values <0.02) and consistently higher than the ones of EMS. Raters appeared to use top-down information to recover underlying messages, resulting in saturated scores near 1.0 and low-to-moderate inter-rater agreement.

**Conclusion:**

Collected remotely with minimal burden, digital speech measures like intelligibility may serve as objective, sensitive outcomes for communication-focused interventions and PSP trials.

## 1 Introduction

Progressive supranuclear palsy (PSP) is a primary four-repeat (4R) tauopathy and the most common form of atypical parkinsonism (Hoglinger et al., 2017). Speech, language, and swallowing impairments are frequent in PSP and contribute substantially to functional decline and reduced quality of life (Hoglinger et al., 2017; Williams and Lees, 2009). At CurePSP’s Patient-Focused Drug Development meeting, speech difficulty was among the symptoms most often identified as affecting daily life (CurePSP, 2026). Therapeutic options nonetheless remain limited: no agent has been shown to alter disease trajectory, and available symptomatic treatments provide only modest and often transient benefits (Coughlin and Litvan, 2022; Lozupone et al., 2024). Contributing factors include intervention late in the disease course, the absence of sensitive biomarkers for early diagnosis and target engagement, and difficulty measuring clinically meaningful change (Coughlin and Litvan, 2022; Lozupone et al., 2024).

PSP speech impairment encompasses a mixed dysarthria with hypokinetic, spastic, and ataxic components, apraxia of speech, and/or nonfluent/agrammatic aphasia (Rusz et al., 2015; Skodda et al., 2011; Kang et al., 2023; Tykalova et al., 2017). Communication management is staged, relying on compensatory and behavioral strategies early and on augmentative and alternative communication as speech becomes unintelligible. Because these deficits track disease progression and carry substantial functional consequences (Garcia-Cordero et al., 2025), their early detection and systematic monitoring are important for guiding clinical management, informing prognosis, and supporting clinical trials with meaningful endpoints.

Digital speech analysis enables automated, reproducible quantification of acoustic, prosodic, and timing features, including articulation rate, pause structure, and voice quality, which are conventionally graded through subjective perceptual ratings at in-person visits. We have previously shown that such measures can differentiate PSP from Parkinson’s Disease (PD) with above 90% accuracy (Kang et al., 2023), separate PSP from Huntington’s Disease (HD), Amyotrophic Lateral Sclerosis (ALS) and healthy elderly controls (Kang et al., 2025), and exhibit more sensitivity to changes in disease severity over time compared to clinical scales such as bulbar score (Kang et al., 2026). Using speech measures as outcomes further requires that each corresponds to an interpretable clinical construct.

Among speech measures, intelligibility is closely tied to functional communication, though the association is moderate rather than deterministic (Borrie et al., 2022). It is defined by what a listener understands, so variation across listeners is intrinsic to the construct rather than incidental to it. In practice, scores depend on listener expertise, familiarity with the speaker, and transcription method (Hustad and Cahill, 2003), and the coarse impressions formed during a clinic visit may miss early change. An automated measure derived from speech recognition output substitutes a fixed algorithmic listener for a human one, removes inter-rater variability and can be applied repeatedly at low burden (Tröger et al., 2024). Establishing what such a measure captures therefore requires explicit comparison against listener-based scoring. Automated intelligibility measures of this kind have been validated mainly in PD and ALS, and PSP has so far been represented only as a small subgroup benchmarked against a clinician-rated speech item (Tröger et al., 2024). In this prospective, cross-sectional study, we provide, to our knowledge, the first validation of a fully-automated digital intelligibility measure in PSP against a composite reference of expert perceptual raters.

## 2 Method

### 2.1 Participants

Forty-nine PSP patients diagnosed according to the 2017 Movement Disorder Society (MDS) criteria were enrolled (see Table 1). The minimum eligibility age for enrollment was 40. Most patients (85%) had the PSP–Richardson syndrome (PSP-RS) phenotype. This is consistent with PSP epidemiology, in which PSP-RS is the most common phenotype (~three-quarters of autopsy-confirmed cases) (Hoglinger et al., 2017). Inclusion criteria included being ambulatory (able to walk at least 10 feet safely without support), able to provide informed written consent, and having a care partner available to support all study-related procedures. Given the low study risk, concurrent enrollment in other studies was permitted, with no prohibited medications.

**Table 1:** Demographic and clinical characteristics of 49 included patients.

Demographics		
Age, years (mean ± SD) [*N* = 33]	68.8 ± 6.6	
Sex, *n* (%) [*N* = 33]	Male	19 (57.6%)
	Female	14 (42.4%)
Race × Ethnicity, *n* (%) [*N* = 33]	Caucasian, Non-Hispanic	22 (66.7%)
	Caucasian, Ethnicity not reported	8 (24.2%)
	African–Black, Non-Hispanic	1 (3.0%)
	Asian–East, Ethnicity not reported	1 (3.0%)
	Caucasian, Hispanic	1 (3.0%)
Total education, *years* (mean ± SD) [*N* = 24]	17.2 ± 2.3	
Employment status, *n* (%) [*N* = 24]	Retired for other reasons/Reached retirement age	14 (58.3%)
	Retired due to medical reasons	6 (25.0%)
	Currently working	3 (12.5%)
	Disabled, permanently or temporarily	1 (4.2%)
Handedness, *n* (%) [*N* = 24]	Right	22 (91.7%)
	Left	2 (8.3%)
Native English-speaker, *n* (%) [*N* = 49]	46 (93.9%)	
Clinical characteristics		
Phenotype, *n* (%) [*N* = 40]	Probable PSP-RS	32 (80%)
	Probable PSP-P	2 (5.0%)
	Possible PSP-RS	2 (5.0%)
	Probable PSP-PGF	1 (2.5%)
	Possible PSP-SL	1 (2.5%)
	Possible PSP-CBS	1 (2.5%)
	Probable PSP-F	1 (2.5%)
Dysarthria severity, score, *n* (%) [*N* = 31]	None (0)	6 (19.4%)
	Minimal (1)	16 (51.6%)
	Moderate (2)	7 (22.6%)
	Severe (3)	2 (6.5%)
Time since diagnosis, months (mean ± SD) [*N* = 31]	18.8 ± 13.0	
Time since symptom onset, months (mean ± SD) [*N* = 21]	46.9 ± 15.4	
Patient reported outcomes		
CBFS speech item (A1), score, *n* (%) [*N* = 33]	None (0)	7 (21.2%)
	Minimal (1)	10 (30.3%)
	Moderate (2)	12 (36.4%)
	Severe (3)	4 (12.1%)
PSP-QoL speech item (11), score, *n* (%) [*N* = 33]	None (0)	9 (27.3%)
	Minimal (1)	8 (24.2%)
	Moderate (2)	8 (24.2%)
	Severe (3)	6 (18.2%)
	Extreme (4)	2 (6.1%)

*n* is the count per parameter, *N* is the total datapoints available for a parameter type. PSPRS, PSP Rating Scale; PSP-RS, PSP Richardson’s Syndrome; PSP-P, PSP with predominant Parkinsonism; PSP-PGF, PSP with Progressive Gait Freezing; PSP-SL, PSP with predominant Speech/Language disorder; PSP-OM, PSP with predominant Ocular Motor dysfunction; PSP-F, PSP with predominant Frontal presentation; PSP-CBS, PSP with predominant Corticobasal Syndrome; CBFS, Cortical Basal ganglia Functional Scale; QoL, Quality of Life.

The study protocols were reviewed and approved by the Mass General Brigham Human Research Committee (2023P001384) and Johns Hopkins Medicine Institutional Review Board (00341607). All procedures were conducted in accordance with the 1975 World Medical Association Declaration of Helsinki, as revised in 1983 (Johns Hopkins University IRB: 00341607; Mass General Brigham IRB: 2023P001384; ClinicalTrials.gov identifier: NCT05956834). Informed consent was obtained from all participants. Study procedures were recorded on ClinicalTrials.gov (NCT05956834). De-identified data that supports the findings of this study are available only for non-commercial applications upon reasonable request.

### 2.2 Data collection

Clinical severity was assessed using dysarthria score (the dysarthria item of the PSP Rating Scale (PSPRS)), as well as patient-reported outcomes including the Cortical Basal ganglia Functional Scale (CBFS) with the sub-item A1 (CBFS-A1) and the PSP Quality of Life (QoL) with the sub-item 11 (PSP-QoL-11) evaluating speech function. For all clinical scales, higher scores indicate more speech impairment.

Table 1 reports the number of available datapoints for each parameter type. Missing data in patient-reported outcomes correspond to self-report burden instances such as fatigue or reduced stamina, apathy, bradyphrenia, and cognitive or communication impairment that make self-rating difficult, absence of a caregiver to help when the patient could not complete items independently, skipped or partially answered items, and instances where the electronic or paper instrument was simply not administered or not returned. Missing data in clinician-administered measures such as the dysarthria score stem from limited appointment time or workflow, telehealth or hybrid visits at which an oral-motor/speech examination could not be reliably conducted, missed or rescheduled in-person visits, or the rater leaving an item unscored (for example, when severe disease prevented the patient from attempting the task). We used all collected data that were available, with each analysis drawing on every participant for whom the relevant score was present.

At a single session, each participant completed the standardized passage-reading task on the BioDigit Clinic™ tablet (BioSensics LLC, Newton, MA, United States) (Kang et al., 2025). The resulting corpus of speech samples was independently rated by three blinded speech-language pathology (SLP) experts and also scored by BioDigit Speech, as detailed below. Digital measurements were compared with the aggregate reference standard from experts.

### 2.3 Reference standard

Three SLPs served as independent expert raters. Each met all of the following criteria:

- Current licensure/registration as an SLP in their jurisdiction.
- Minimum 5 years of post-fellowship clinical experience with motor speech disorders.
- Completion of rater training prior to data collection.

All three raters were blinded to the following throughout the rating phase:

- BioDigit Speech scores for any sample.
- Ratings provided by the other two raters.
- Severity stratum assignment of samples.
- Any demographic or clinical information about the speaker beyond what is audible in the recording.

Raters assigned an intelligibility score from 0 to 100 (integer). All ratings were conducted in a quiet environment. Each rater listened to each sample no more than two times, and estimated intelligibility as the percentage of correctly-understood words among the total number of words. Raters did not use transcripts or any contextual information that is not present in the audio. Rater scores were divided by 100 for consistency of comparison with digital intelligibility at 0–1 scale.

The primary reference standard against which BioDigit Speech scores were compared was the mean of the three expert SLP ratings for each sample (Expert Mean Score: EMS). As the perceptual rating itself varies across raters, it is best characterized as a range of ratings, operationalized here as EMS. This choice is supported by prior evidence. (Dahl et al., 2025) showed that 3–4 is an ideal number for perceptual ratings. Whitehill, 2002 showed that single-judge intelligibility ratings carry substantial random error and advocated composite ratings for research measurement. Hustad, (2008) used mean listener ratings as the validity criterion in dysarthria research for the same reason.

### 2.4 Software scoring procedure

Speech data were processed and analyzed using BioDigit Speech™ (BioSensics LLC, Newton, MA, United States) (Kang et al., 2025). BioDigit Speech uses automated speech recognition (ASR) based on WhisperX (Bain et al., 2023) to transcribe participant speech and generate word-level timestamps. Intelligibility represents the word-level agreement between the transcriptions produced by the medium version of the WhisperX model and by the tiny version; both versions were developed for the English language. The rationale behind comparing the outputs from models of different complexities is that the smaller model struggles to accurately transcribe unclear speech. Consequently, the less intelligible the speech, the lower the accuracy of the smaller model relative to the larger model, and the lower the resulting intelligibility score.

Word-level agreement is a continuous measure on a scale of 0–1, computed as the complement of Word Error Rate (WER) in the transcription by the smaller model compared to the transcription by the larger model. The two transcriptions are standardized first by unifying letter cases, and removing punctuations and a/an/the articles. Standardized transcriptions are aligned word-by-word using the minimum-edit (Levenshtein) alignment, which counts three kinds of errors: substitutions (a word from the larger model replaced by a different word by the smaller model), deletions (a word from the larger model missing from the smaller model), and insertions (an extra word from the smaller model not generated by the larger model). WER is the total number of these errors divided by the number of words in the larger model’s transcription; 1 − WER then gives the digital intelligibility score. As insertion errors do not have a known limit, lowest intelligibility value is saturated at 0 to match the human perceptual rating scale.

BioDigit Speech processed each speech sample independently using the same audio files presented to human raters (no pre-processing differences). Intelligibility scores were generated in batch mode and segregated from expert ratings until the database lock. The software operator was blinded to expert ratings at the time of scoring. As intelligibility calculation relies on ASR, accent differences can degrade the accuracy of generated scores, motivating the subgroup analysis with native English-speakers (see Section 3).

### 2.5 Quality control

Prior to scoring, all speech recordings underwent manual quality screening; all samples passed quality control. Quality review included checks for truncated or incomplete audio, clipping or saturation consistent with microphone or hardware failure, and silence or non-speech audio (e.g., only ambient noise or study coordinator speaking). This mirrored the quality control screening in our completed PSP-RS studies (Kang et al., 2025; Kang et al., 2026).

### 2.6 Statistical analysis

Agreement between the BioDigit Speech software and EMS was evaluated using the consistency, average-measures intraclass correlation coefficient [ICC(C,k)] (Koo and Li, 2016), and their association was evaluated using Pearson correlation. The ICC was calculated using all samples, along with a 95% confidence interval (CI). A Bland–Altman plot (Bland and Altman, 1986) was also generated for difference scores (Software−EMS) vs. the mean of software and EMS. The following statistics were reported:

- Mean bias (d̅): systematic over- or under-estimation of EMS by the software.
- Standard deviation of differences (SD).
- Regression-based (sloped) Limits of Agreement (LoA) (Koo and Li, 2016).
- 95% CI for d̅ and each LoA.
- Proportional bias: linear regression of the difference scores on their means to test whether agreement varies systematically across the intelligibility range.

ICC was also used to evaluate inter-rater agreement.

We quantified the discrimination ability of digital intelligibility vs. EMS for each clinical scale (dysarthria severity, CBFS-A1 and PSP-QoL-11). Discrimination of each ordinal clinical score by the two intelligibility measures (EMS vs. digital) was quantified with rank concordance using Somers’ D and the equivalent c-index (Somers, 1962; Harrell, 2015; Harrell et al., 1982; Newson, 2006; Kendall, 1938; DeLong et al., 1988). Computations were over all comparable participant pairs differing on the clinical score, with predictor ties weighted by 0.5. Larger absolute values |D| (c-index farther from 0.5) indicates better separation of the score levels. Because the two intelligibility measures were evaluated on the same participants, their discrimination indices are statistically dependent; the absolute change in discrimination, Δ|D| = |D(digital)| − |D(EMS)|, was therefore tested with a subject-level bootstrapping. The two-sided percentile bootstrap *p*-value was computed as 2 × minimum [P(Δ|D| ≤ 0), P(Δ|D| ≥ 0)], where P indicates probability. CIs derived by bootstrapping used the same nonparametric, participant-level scheme in both regression-based LoA and |D| analyses (Efron, 1979; Efron and Tibshirani, 1993; Davison and Hinkley, 1997): participants were resampled with replacement, the statistic of interest was recomputed on each resample, and 95% CIs were taken as the 2.5th and 97.5th percentiles of the resampled distribution.

As complementary descriptors, we also report a standardized slope for each measure: each measure was standardized to z-scores using the sample SD with 1 degrees of freedom and then regressed on the raw ordinal clinical score by ordinary least squares (OLS), so the slope is expressed in SD of the measure per one-point increase in the score. To test whether the two measures changed at different rates across the score, we computed a paired within-subject contrast: the within-participant difference of the standardized measures (z(digital) – z(EMS)) was regressed on the clinical score by OLS, and the two-sided *p*-value for this contrast was the t-test on that regression slope. This contrast slope is algebraically equal to the difference in the two measures’ standardized slopes; a negative reported value indicates that the digital measure had the more negative standardized slope, i.e., it decreased more steeply or changed more per one-point increase in the clinical score, and a nonzero contrast slope indicates that the two standardized measures responded to the score at significantly different rates. Finally, Pearson correlations between each intelligibility measure and each clinical scale were also computed for descriptive comparison.

## 3 Results

Figure 1 shows correlations of EMS with digital measurement of intelligibility, separately for all participants and native English-speaking participants. EMS showed high correlation with digital intelligibility (*r* = 0.88 for all participants and *r* = 0.90 for native English-speaking participants). Rater scores were saturated close to 1.0, and showed low-to-moderate inter-rater agreement with pairwise rater ICCs of 0.48, 0.68, and 0.77. For participants with lower digital intelligibility measures, the ranges of ratings among the three raters are also shown in horizontal error bars in Figure 1, further highlighting inter-rater variability.

**Figure 1:**
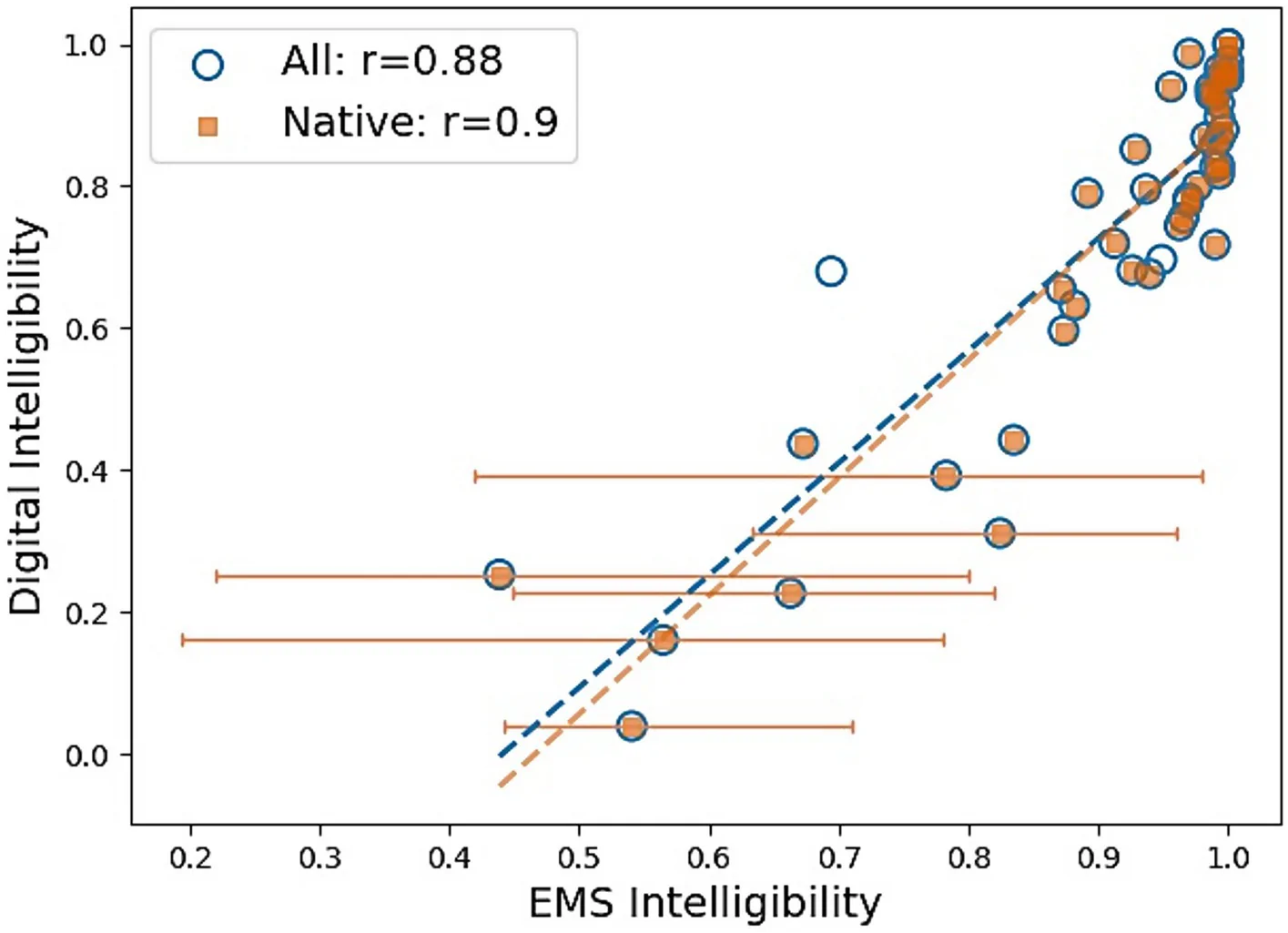
Correlations of EMS with digital measurement of intelligibility for all vs. native English-speaking participants. For participants with lower digital intelligibility measures, the ranges of ratings among the three raters are shown in horizontal error bars.

Figure 2 shows Bland–Altman and ICC results comparing digital intelligibility and EMS for native English-speaking participants. Agreement between the digital and expert (EMS) intelligibility scores was high in consistency (ICC[C,k] = 0.84, 95% CI: 0.71–0.91). On average, the digital score fell below EMS: mean bias (digital − EMS) = −0.18 (95% CI: −0.23 to −0.14), with SD of the differences = 0.16 (95% CI: 0.13 to 0.20). The difference increased with the magnitude, indicating statistically significant proportional bias (slope = 0.68, 95% CI: 0.55 to 0.81; *r* = 0.85; *p* < 0.0001); fixed 95% LoAs were therefore not valid and regression-based LoAs were reported instead. These were given by bias (A) = −0.74 + 0.68·A (bootstrap 95% CI for the intercept: −0.97 to −0.55; for the slope: 0.47 to 0.94), with a non-uniform scatter model (SD of the differences increasing with the mean), so the limits widened and shifted downward toward lower intelligibility. As only three participants were non-native English speakers, including them did not change the results substantially (e.g., ICC(C,k) = 0.840 for native English speakers vs. 0.837 for all participants).

**Figure 2:**
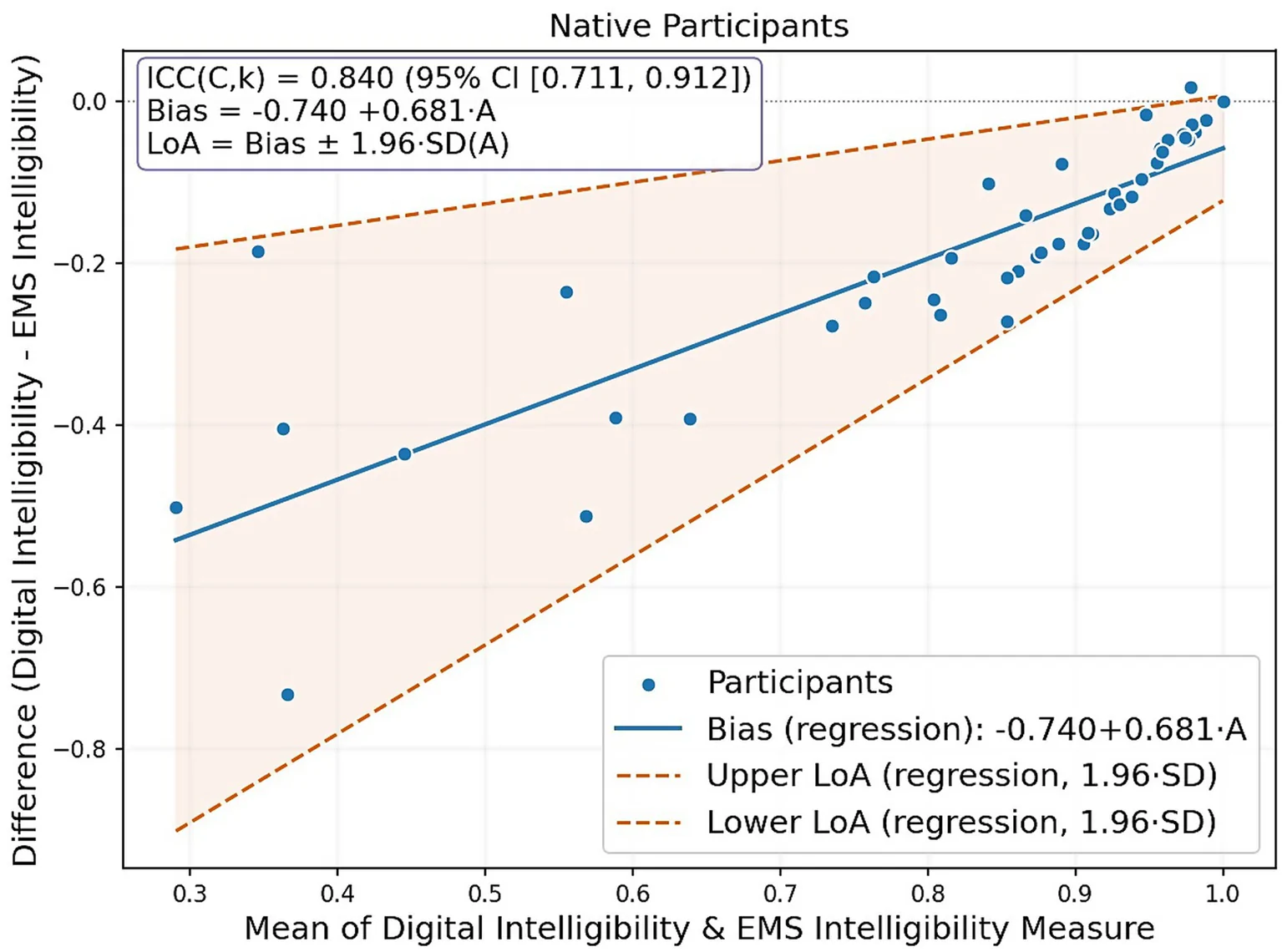
Bland–Altman and ICC results for comparing digital intelligibility and EMS for native English-speaking participants.

Figures 3A–C show digital intelligibility vs. EMS grouped by PSP-QoL-11, dysarthria severity and CBFS-A1 scores for native English-speaking participants. The digital measure discriminated PSP clinical severity as well as or better than expert EMS ratings. For PSP-QoL-11, the digital measure was nominally stronger (|D| = 0.48 vs. 0.44); although the difference in Somers’ D was not significant (Δ|D| = +0.033, bootstrap *p* = 0.69), the paired standardized-slope contrast was (difference in standardized slope = −0.14, *p* = 0.018), indicating that the digital score changed more steeply per unit of PSP-QoL-11. For dysarthria severity, the two measures were essentially equivalent (|D| = 0.43 vs. 0.43; c-index = 0.29 vs. 0.29; Δ|D| = +0.003, bootstrap *p* = 0.98; contrast *p* = 0.22), while for CBFS-A1, EMS was nominally stronger (|D| = 0.56 vs. 0.47) but not significantly so (Δ|D| = −0.089, bootstrap *p* = 0.31; contrast *p* = 0.62). We also computed Pearson correlations between the clinical scales and the two intelligibility measures for native English-speaking participants. Digital intelligibility correlated more strongly with each clinical scale than EMS (*r* = −0.49 vs. −0.32 for PSP-QoL-11, *r* = −0.51 vs. −0.47 for CBFS-A1, and *r* = −0.42 vs. −0.32 for dysarthria severity); all correlations of digital intelligibility with the clinical scales were significant (*p* < 0.02).

**Figure 3:**
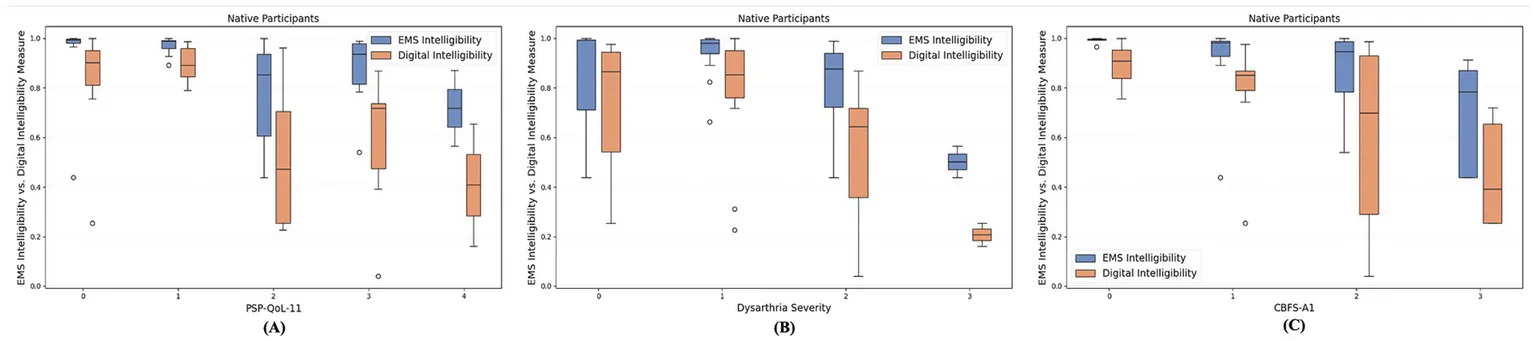
**(A)** Digital intelligibility vs. EMS grouped by PSP-QoL-11 scores for native English-speaking participants. **(B)** Digital intelligibility vs. EMS grouped by dysarthria severity for native English-speaking participants. **(C)** Digital intelligibility vs. EMS grouped by CBFS-A1 scores for native English-speaking participants.

## 4 Discussion

EMS and digital intelligibility showed high correlation and agreement (*r* = 0.90, ICC = 0.84 in native English speakers). Where the two diverge, digital intelligibility systematically scored below EMS and the discrepancy was severity-dependent: negligible for near-normal speech but large for impaired speech. The observed bias may thus be addressed through regression-based calibration, particularly at the low-intelligibility end, although any such calibration would need to be derived and validated in an independent sample. One plausible explanation for the tendency of BioDigit Speech to score below EMS is that the algorithm registers subtle and residual speech deviations that human listeners compensate for by using top-down linguistic knowledge to recover the intended message, resulting in perceptual scores saturated near 1 with low-to-moderate inter-rater agreement. However, the present design cannot distinguish this greater resolution from systematic underestimation by the algorithm (for example, transcription errors of the smaller ASR model that are unrelated to perceived intelligibility), and this interpretation remains to be tested against an independent external criterion. The strong agreement of EMS and digital intelligibility despite only low-to-moderate agreement among individual raters also suggests that averaging across the three experts cancels idiosyncratic rater error and isolates the shared, reliable intelligibility signal that the software tracks (Nunnally and Bernstein, 1994).

Further, the digital measure matched or exceeded EMS in distinguishing PSP clinical severity (most notably for PSP-QoL-11, where it showed a steeper standardized response) and correlated more strongly with the clinical scales than EMS. The natural next question of whether the system can separate healthy individuals from patients with early or suspected PSP relates to our prior work, which showed that digital measure of intelligibility can separate PSP from healthy elderly controls and PD (Kang et al., 2025) and exhibit more sensitivity to changes in disease severity over time compared to clinical scales such as the bulbar score (Kang et al., 2026). Whether this extends to early or suspected PSP remains to be established in prospective diagnostic cohorts, and that such use would require a diagnostic-accuracy validation framework distinct from the analytical validation reported here. Overall, an analytically valid, longitudinally sensitive digital measure such as intelligibility could serve as an objective, patient-relevant endpoint that complements existing clinical scales, enabling earlier detection of communication decline, more sensitive tracking of disease progression and treatment response, and timelier initiation of speech-language therapy and augmentative/alternative communication, thereby supporting both PSP clinical management and trial enrichment and outcome assessment.

Our findings can be situated within a growing set of automated intelligibility measures. Moya-Galé et al. validated an automatic estimate of intelligibility-in-noise for PD (Moya-Galé et al., 2022). Tröger et al. developed an ASR-derived intelligibility metric validated across multiple languages and neurological disorders (Tröger et al., 2024) that subsequently modeled bulbar-specific severity and progression in ALS (Tröger et al., 2026). Stegmann et al. tracked bulbar decline in ALS from frequent, remotely collected speech (Stegmann et al., 2020). BioDigit Speech differs from these efforts in three respects that bear on its interpretation. First, the multi-disorder validation of ASR-based intelligibility (Tröger et al., 2024) was benchmarked against clinician-rated speech items rather than perceptual intelligibility, and comparisons against listener transcription have been reported mainly in PD (Moya-Galé et al., 2022). Instead, we validated digital intelligibility directly against a composite, multi-rater expert intelligibility reference, which let us characterize where the algorithmic and human listeners diverge rather than only whether they covary. Second, while ASR-based intelligibility scores have typically been computed against a known target text (Tröger et al., 2024; Moya-Galé et al., 2022), our measure quantifies agreement between two ASR models of different capacity and therefore does not require a reference transcript, which in principle extends it to unscripted speech. Third, although PSP was represented in the multi-disorder validation (Tröger et al., 2024), that subgroup was small (*n* = 17) and its association with the clinician-rated speech item reached only trend-level significance. To our knowledge, the present study is the first to validate automated intelligibility in PSP against expert perceptual ratings, and it does so in a substantially larger PSP cohort (*n* = 49). The mixed hypokinetic–spastic–ataxic dysarthria of PSP also differs from the profiles of ALS and PD in which these tools were primarily developed. Consistent with the cross-disorder generalization reported by (Tröger et al, 2024), our results indicate that ASR-based intelligibility recovers clinically meaningful variance across etiologies; the systematic, severity-dependent divergence from expert raters that we observed further clarifies that automated and perceptual intelligibility are related but non-identical constructs: a distinction likely shared by, but not previously quantified for, these tools.

All in all, this study extended digital intelligibility measurement that has been validated mainly in PD (Tröger et al., 2024; Moya-Galé et al., 2022) and ALS (Tröger et al., 2026; Stegmann et al., 2020) into PSP, and complements our longitudinal PSP work (Kang et al., 2026) by supplying the agreement-with-experts foundation such progression-tracking claims depend on. Potential limitations include rater familiarity with the standardized passage: because all participants read the same text, repeated exposure across samples may have inflated perceptual intelligibility judgements (Hustad and Cahill, 2003). Related to this, EMS remains a subjective measure and is affected by inter-rater variability and potential ceiling effects. Therefore, the current study primarily demonstrates agreement and convergent validity with expert perceptual ratings rather than validation against an independent objective criterion. Moreover, for the few non-native speakers, both digital and EMS-based intelligibility measures may have been affected by accent differences. In addition, missing data in clinical scores may have affected the precision of findings on the relationship between clinical scores and digital measures, as using only available paired data reduces the effective sample size. Further, the sample was predominantly PSP-RS and Caucasian with high educational attainment, and demographic and clinical data were incomplete for a fraction of participants, which limits generalizability. Future work will involve longitudinal validation of multimodal measurements of speech function measured by BioDigit Speech, including other disease populations such as HD.

## Data Availability

The datasets presented in this article are not readily available because De-identified data that supports the findings of this study are available only for non-commercial applications upon reasonable request. Requests to access the datasets should be directed to Ashkan Vaziri, ashkan.vaziri@biosensics.com.
